# *Ossa cordis* and os aorta in the one‐humped camel: Computed tomography, light microscopy and morphometric analysis

**DOI:** 10.1002/jemt.24256

**Published:** 2022-11-10

**Authors:** Samir A.A. El‐Gendy, Mohamed A.M. Alsafy, Catrin S. Rutland, Samar M. Ez Elarab, Hanan H. Abd‐Elhafeez, Basma M. Kamal

**Affiliations:** ^1^ Department of Anatomy and Embryology, Faculty of Veterinary Medicine Alexandria University Alexandria Egypt; ^2^ School of Veterinary Medicine and Science, Faculty of Medicine University of Nottingham Nottingham UK; ^3^ Department of Histology and Cytology, Faculty of Veterinary Medicine Alexandria University Alexandria Egypt; ^4^ Department of Cell and Tissues, Faculty of Veterinary Medicine Assiut University Asyut Egypt; ^5^ Department of Anatomy and Embryology, Faculty of Veterinary Medicine University of Sadat City Sadat Egypt

**Keywords:** camel, computed tomography, heart, light microscopy, os aorta, *Ossa cordis*

## Abstract

The present study describes the morphological characteristics of the camel heart *Ossa cordis*, and os aorta using computed tomography soft tissue window (CT) alongside 3D render volume reconstructions and light microscopy. The current study techniques demonstrated the *Ossa cordis* and os aorta in the cardiac window with more precision than the black and white (ghost), and angiography images. Transverse and sagittal CT images additionally demonstrated the presence of *Ossa cordis* and os aorta. This study is the first to record two small *Ossa cordis sinistrum* and one os aorta in the camel heart, in addition to the more commonly observed singular, large, os cordis dextrum. The os cordis dextrum was always located in the upper part of the interventricular septum, near to its junction with the atrium, forming an elongated rectangular shape when observed transversally. The wider cranial part was composed from bone, whereas the caudal aspect was narrow and contained both bone and cartilage. Light microscopy identified that the os cordis dextrum consisted of trabecular bone, marrow spaces, and hyaline cartilage. Two *Ossa cordis sinistrum* were detected on the left side of the heart, one in the right fibrous ring and another in the interventricular septum, microscopy showed that both contained only trabecular bone with osteocytes, osteoblasts, and osteoclasts. At the level of ascending aorta, there was also trabecular bone containing osteocytes, an os aorta.

## Graphical abstract

*Ossa cordis* and os aorta in the camel.

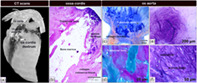

**Research Highlights**

- Discovery of os cordis dextrum and two *Ossa cordis sinistrum* in the camel.
- Discovery of os aorta in addition to *Ossa cordis* in camels.
- Combined use of computed tomography and light microscopy to analyze *Ossa cordis* and os aorta.

## 1 INTRODUCTION

The dromedary camel (*Camel dromedarius*) is extremely well adapted to life in hot and arid lands. Dromedary camels have very special anatomical and physiological characteristics, which enables these animals to live, reproduce, produce milk, and meat, and to thrive under these extreme heat and arid conditions, even during times of drought when cattle, sheep, and goats may fail to survive (Bornstein, 1990). They are an important sustainable livestock species in many parts of the world, in addition to being used for transportation, in tourism industries and sports, camels have even been declared as national and state animals in many regions. Modern camels are in the Artiodactyla order, within the *Tylopoda* suborder. The Camelini (Old World camels) and Lamini (New World camels) tribes diverged around 16.3 million years ago (Wu et al., 2014). Although camels are ruminating animals, they are not within the *Ruminantia* suborder, due to differences in their stomach system, foot anatomy and because they have no horns (Wernery, 2003). The hearts of Old World camels and New World camels also have more reported similarities with each other than with domestic ruminants (Perez et al., 2018).

Within the heart, the cardiac skeleton (*anulus fibrosus*) contains four rings of dense fibrous connective tissue into which heart muscle fibers are inserted, helping provide structure for the heart alongside isolating electrical signaling between the ventricles and atria (Drake et al., 2009). This important tissue also supports and anchors the heart valves thus helping to keep the atrioventricular and semilunar valves open. The cardiac skeleton also contains two fibrous trigones, the thickest structures within the cardiac skeleton, and a ligament, in addition to other structures. The left fibrous trigone (trigonum fibrosum sinsitrum) surrounds the bicuspid valve whereas the right fibrous trigone (trigonum fibrosum dextrum) encircles the tricuspid valve, with the membranous septum creating extensions of the cardiac skeleton into the interatrial and interventricular septae (Balah et al., 2014; Dellmann & Eurell, 1998; Gartner & Hiatt, 2007; Ghallab, 2000). In a limited number of mammalian species, the trigones may contain fibro‐cartilage, hyaline cartilage (cartilago cordis), and even bone (on the right or left, os cordis dextrum and os cordis sinistrum respectively). *Ossa cordis* can expand into the trigone(s) between the atrioventricular openings and therefore the aortic opening. Previous work in differing species has shown that *Ossa cordis dextrum* are more common, and are usually larger, than those on the left (Best et al., 2022).

The presence of *Ossa cordis* have been identified in a few species, including (but not limited to) cattle, sheep, goats, pigs, otters and chimpanzees (Egerbacher et al., 2000; Malik et al., 1972; Mia, 1973; Moittie et al., 2020; Nickel et al., 1995; Schmack, 1974). A comprehensive review of all species and potential functions is available (Best et al.). The exact functions of *Ossa cordis* are still debated; however, they may help provide structure and support, and may assist with electrical conduction in the heart (Best et al., 2022). In the chimpanzee the presence of *Ossa cordis* or *Cartilago cordis* was linked to disease progression and age, indeed age is a contributing factor in a number of species including sheep and otters. Increases in calcium, phosphorus, and magnesium in the right and left fibrous trigones have also been observed in the aging human (Tohno et al., 2007). The developmental processes by which *Ossa cordis* develop are also debated, with some evidence linking ossification of cartilage to the process, such as that reported in due to advancement of age in sheep, goats, and otters (Aretz, 1981; Egerbacher et al., 2000; Frink & Merrick, 1974). Both *Ossa cordis* and *Cartilago cordis* can vary greatly in size, shape, and position between species and even within species. The *Cartilago cordis* also exists within species, which do not develop *Ossa cordis*, and it is also not linked to factors such as body size, taxonomic relationships, or habitat preference (Young, 1994).

Despite these earlier reports describing the basic morphology of the heart and the histology of the single os cordis in the dromedary camel, studies have not been conducted using detailed imaging such as CT or 3D CT. Therefore, this study investiagted *Ossa cordis* within the dromedary camel heart (*Camelus dromedarius*) using CT, 3D CT and histological examinations to provide a detailed study on *Ossa cordis*. The present work also discovered that camels can have not just a singular os cordis dextrum, as previously stated, but could also have two smaller *Ossa cordis sinistrum* and one os aorta.

## 2 MATERIALS AND METHODS

Hearts from five adult, male, healthy camels aged 13 years old, with no history of clinical cardiac abnormalities were slaughtered by a professional veterinary surgeon. The camels were not slaughtered for research purposes, permission was granted to work on the hearts. The work was approved by the Animal Welfare and Ethics Committees, in the Faculty of Veterinary Medicine, Alexandria University and the School of Veterinary Medicine and Science, University of Nottingham (No. 3524 211,209), in accordance with institutional, national and international guidelines. The hearts were dissected out at the slaughterhouse, placed on ice, and immediately transferred to the laboratory. The hearts were examined and imaged within 2 hours of death in order to reduce any potential postmortem variations. Immediately following CT imaging, the specimens were processed for histology.

### 2.1 Computed tomography (3D render volume CT; 128‐slice multi‐detector CT scanning protocol)

Each camel heart was positioned on its right surface for each scan. The hearts were wrapped in a secure in foam material to avoid any movement that would interfere with image quality. The CT X‐ray examinations were performed on an AMDCT scanner with 128 detectors (Aquilion; Toshiba Medical Systems, Tokyo, Japan), with a rotation time of 300 ms, and a slice collimation of 128 × 0.6 mm^2^, using a continuous helical scan mindose technique. After obtaining a preliminary image to determine the 3D‐CTA scan range, serial cross section scans from the apex to the base of each heart were conducted using a slide thickness of 1 mm with 1.3 mm intervals (in accordance to other values of measurements, sections did not overlap but several scans were undertaken), with the following soft window settings: mAs 240, Kv 130, W.342 L.52.

To obtain sagittal sections using the soft tissue window, the scans were conducted at the level of the interventricular septum and about 20 scans were made with 1.8 mm intervals at a thickness of 8 mm, with setting set at: mAs 240, Kv 130, W.342 L.52. CT image reconstruction was undertaken using the optimal reconstruction parameters for hearts. Initially, a set of cross‐sectional tomography slices were constructed individually, then the images were stacked together sequentially to obtain a 3D image model of each heart. The reconstruction algorithm was used within octopus software, then converted into a DICOM format producing three reconstruction images: ghost (black and white) render, color render (red in color) and an angiograph which was translucent blue (Dankowski et al., 2014).

The CT images were analyzed using the ImageJ 1.53 k application (National Institutes of Health, USA) to measure the *Ossa cordis* using the previously described method (Alsafy & El‐Gendy, 2022; Moittie et al., 2020; Witkowska et al., 2014).

### 2.2 Staining and microscopy

Following the CT scans, specimens were manually dissected. The three hearts containing *Ossa cordis* dextrum and sinistrum and os aorta, as observed from the CT scans, were processed for histological analysis. These samples were then fixed in 10% buffered neural formalin and decalcified. The samples were decalcified using 10% ethylene damine tetra acetic acid (EDTA) in 0.1 M Tris/HCl buffer, pH 7.4, for 6 days, with solution changes every 2 days (Abd‐Elhafeez et al., 2021). Thereafter the specimens were dehydrated in ethanol, cleared in benzene, then embedded in paraffin wax. Serial sections 5‐7 μm thick were prepared using a Reichert Leica RM 2125 Microtome (Germany), mounted on glass slides, stained with either hematoxylin and eosin (H&E), Safranin O, Crossman's Trichrome, Mallory's trichrome, Toluidine Blue or Alcian blue pH 2.5 (Bancroft & Gamble, 2008), then analyzed using light microscopy.

## 3 RESULTS

### 3.1 Discovery of multiple *Ossa cordis* and os aorta, CT and histological examinations

**FIGURE 1:**
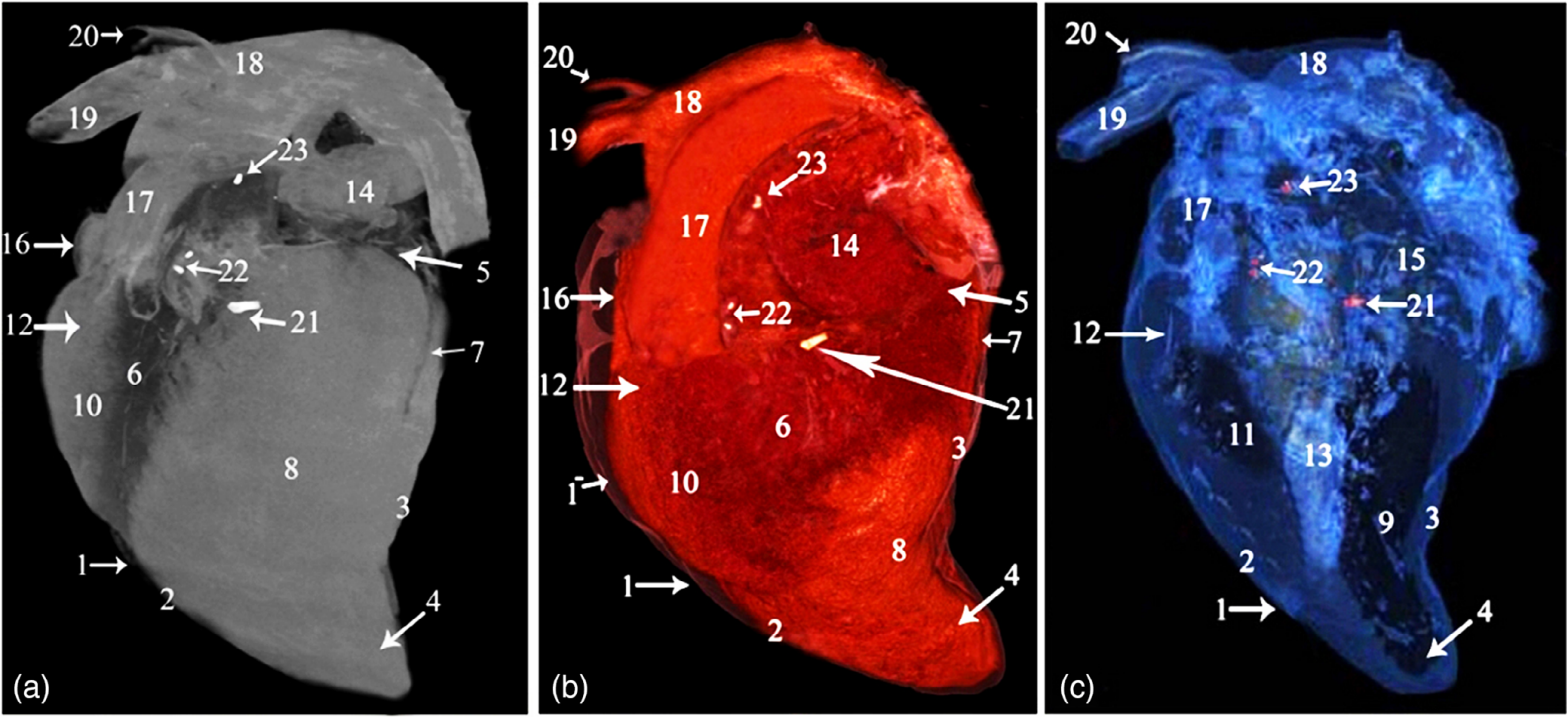
CT 3D render volume of the camel heart showing the *Ossa cordis* and os aorta, left view. Images from (a) ghost (black and white), (b) color render, and (c) blue angiography without contrast media. 1. Pericardium, 1^−^. Pericardial cavity, 2. Cranial border, 3. Caudal border, 4. Apex, 5. Coronary groove, 6. Left longitudinal groove, 7. Intermediate groove, 8. Left ventricle, 9. Left ventricular cavity, 10. Right ventricle, 11. Right ventricular cavity, 12. Conus arteriosus, 13. Interventricular septum, 14. Left auricles, 15. Cavity of the left auricle, 16. Right auricles, 17. Pulmonary artery, 18. Aortic arch, 19. Brachiocephalic trunk, 20. Subclavian artery, 21. *Os cordis dextrum*, 22. *Os cordis sinistrum*, 23. Os aorta

**FIGURE 2:**
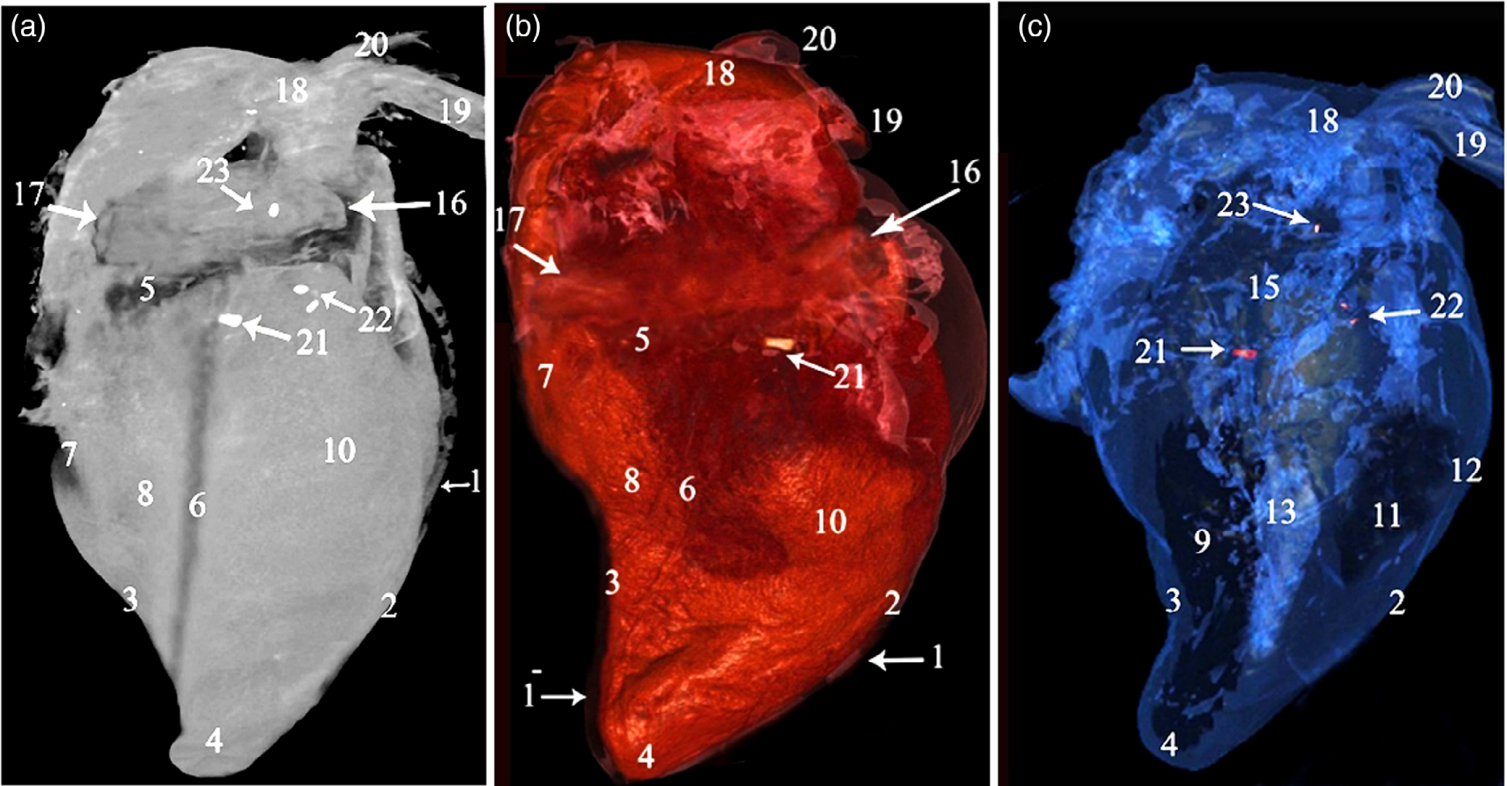
CT 3D render volume of the camel heart, showing the *Ossa cordis* and os aorta, right view. Images from (a) ghost (black and white), (b) color render, and (c) blue angiography without contrast media. 1. Pericardium, 1^−^. Pericardial cavity, 2. Cranial border, 3. Caudal border, 4. Apex, 5. Coronary groove, 6. Right longitudinal groove, 7. Intermediate groove, 8. Left ventricle, 9. Left ventricular cavity, 10. Right ventricle, 11. Right ventricular cavity, 12. Ventricular wall, 13. Interventricular septum, 14. Right atrium, 15. Cavity of the right atrium, 16. Cranial vena cava, 17. Caudal vena cava, 18. Aortic arch, 19. Brachiocephalic trunk, 20. Subclavian artery, 21. *Os cordis dextrum*, 22. *Ossa cordis sinistrum*, 23. Os aorta

**FIGURE 3:**
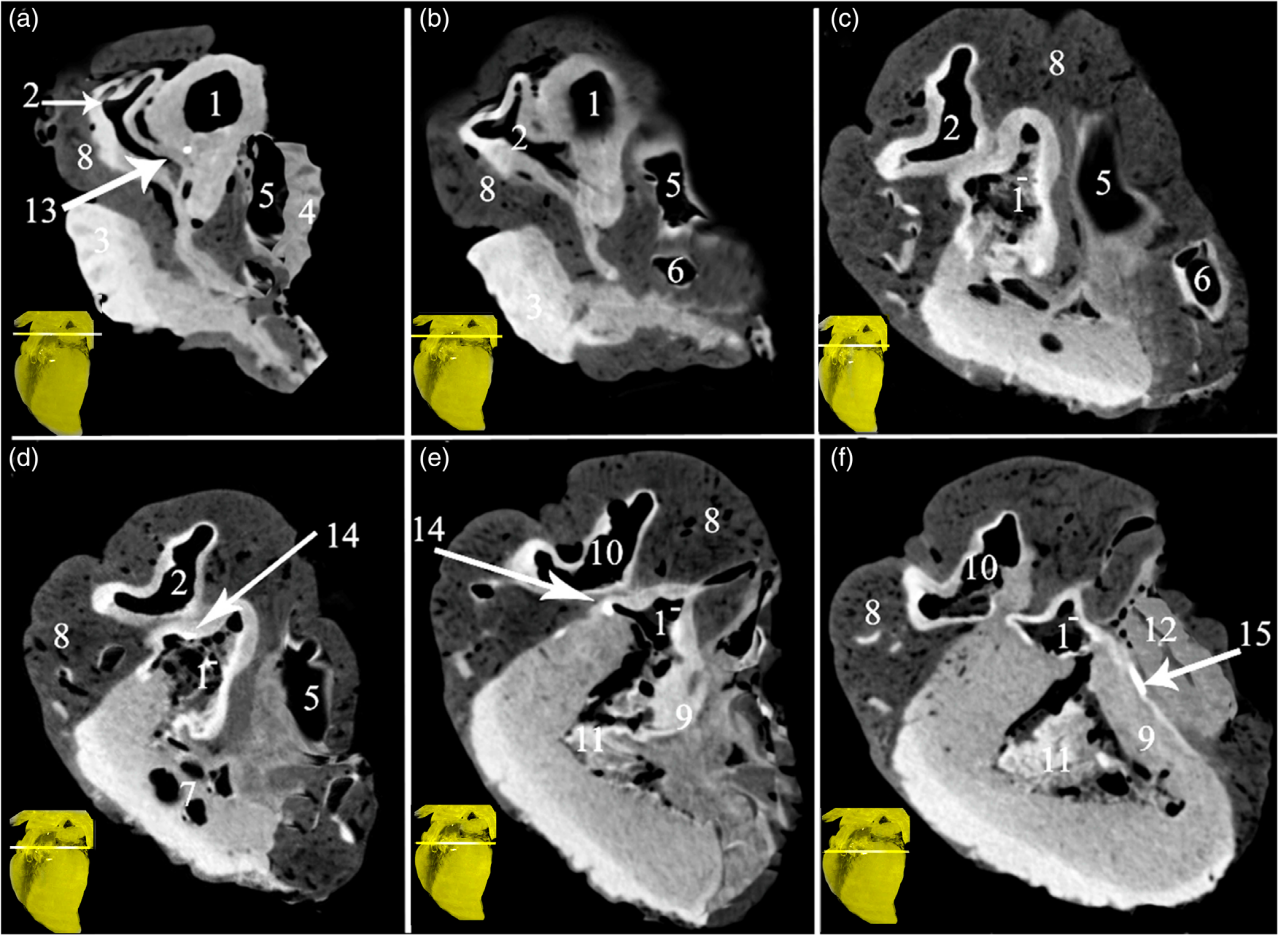
Transverse soft tissue window CT scans at the base (atrial part) of the heart displaying the *Ossa cordis* and os aorta. 1. (A‐F) Scans taken at layers through the heart. Ascending aorta, 1^−^. Aortic orifices (valve), 2. Pulmonary trunk, 3. Left auricle, 4. Right auricle, 5. Cranial vena cava, 6. Caudal vena cava, 7. Pulmonary vein, 8. Subcardinal fat, 9. Interventricular septum, 10. Conus arteriosus, 11. Left atrioventricular opening, 12. Right atrioventricular orifice, 13. Os aorta, 14. *Ossa cordis sinistrum*, 15. *Os cordis dextrum*.

**FIGURE 4:**
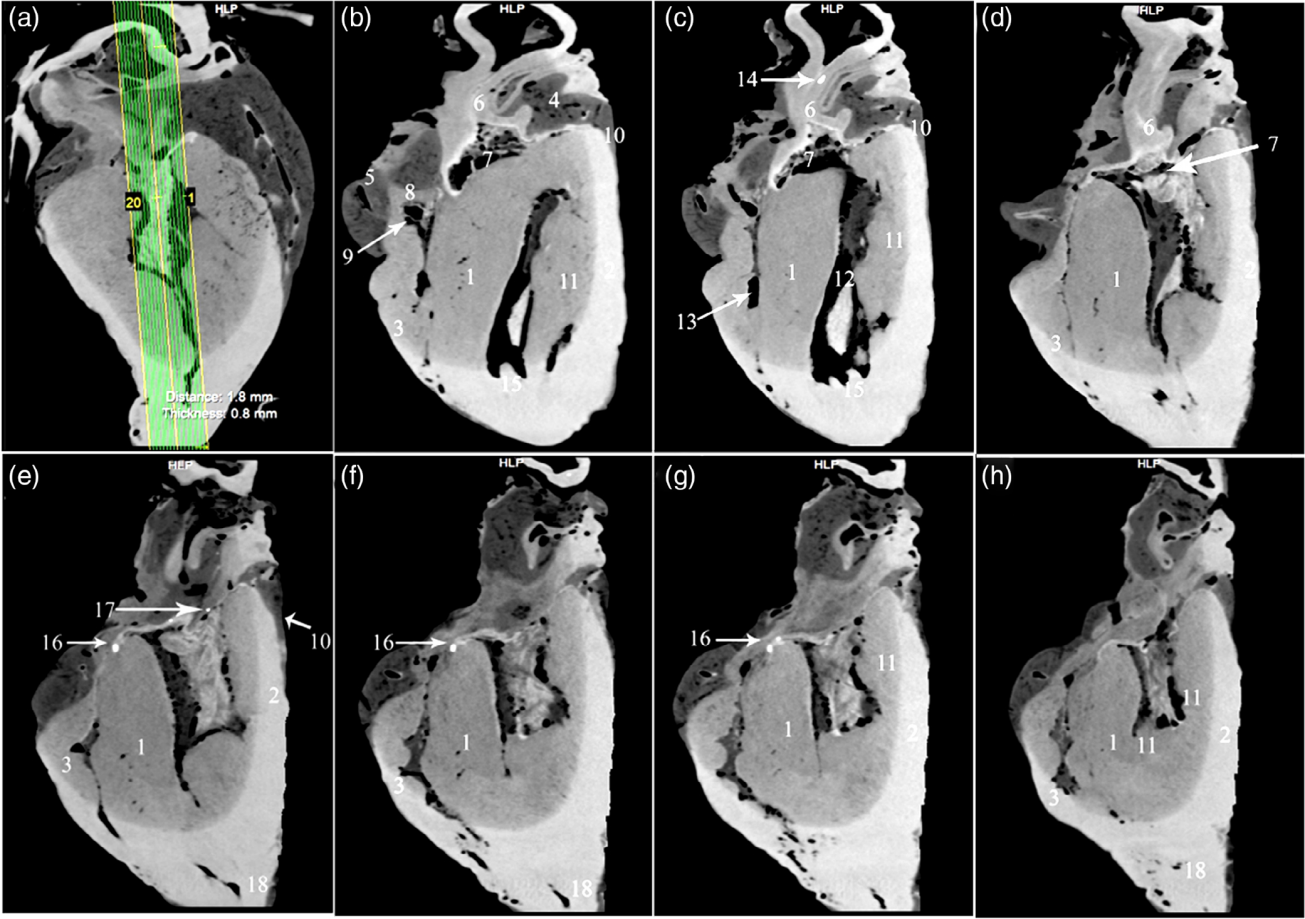
*Ossa cordis* and os aorta in sagittal soft tissue window CT scans, at level of the aorta, in the camel heart. (a) Overview of scan area. (b‐h) scan levels 1, 3, 8, 12, 13, 17, and 20 respectively (termed levels 1–7 in Table 1). 1. Interventricular septum, 2. Mural wall of the right ventricle, 3. Mural wall of the left ventricle, 4. Left atrium, 5. Right atrium, 6. Aorta, 7. Aortic orifice, 8. Right atrioventricular orifice, 9. Chordae tendinae, 10. Subcardinal fat, 11. Papillary muscle, 12 left ventricle, 13. Right ventricle, 14. Os aorta, 15. Trabeculae carneae, 16. *Os cordis dextrum*, 17. *Ossa cordis sinistrum*, 18. Heart apex

Two of the five hearts from male, 13‐year‐old camels each contained a single os cordis dextrum. The remaining three hearts contained one large os cordis dextrum, two smaller *Ossa cordis sinistrum*, and one os aorta in each of the three hearts (Figures 1, 2, 3, 4). The os cordis dextrum was located at the upper part of the interventricular septum near to its junction with the atrium (Figure 3). Each was located transversely and presented with an elongated rectangular shape. In each of the three specimens, the cranial part of the os cordis dextrum was wide and ossified, whereas the caudal part was narrow and contained cartilage. The os cordis dextrum was an average of 12.62 mm long, with a width ranging from 3.96 to 2.84 mm (see Table 1 for all measurements). In each heart, one os cordis sinistrum was detected at the right fibrous ring and another one at the interventricular septum (Figures 1 and 3). The average lengths of the *Ossa cordis sinistrum* ranged between 3.4 and 4.4 mm, whereas the widths ranged from 1.3 to 1.9 mm (Table 1). The os aorta was represented at the wall of the ascending aorta. It measured an average of 3.73 mm long and 2.03 mm wide in the three specimens (Table 1).

**TABLE 1:** *Ossa cordis* and os aorta measurements

	*Ossa cordis dextrum*				*Ossa cordis sinistrum*				Os aorta	
	Length	Width (widest)	Width (mid‐point)	Width (narrow)	#1 length	#1 width	#2 length	#2 width	Length	Width
Heart 1	12.03	4.1	2.87	3.1	3.4	1.39	4.4	1.3	3.4	2.1
Heart 2	13.33	3.9	2.9	3.3	3.8	1.66	3.9	1.7	3.6	1.8
Heart 3	12.5	3.88	2.76	3.4	3.63	1.9	3.95	1.61	4.2	2.2
Average	12.62	3.96	2.84	3.26	3.61	1.65	4.08	1.54	3.73	2.03

*Note*: Measurements in mm.

3D rendered volume of the bones were detected using black and white, color, and blue angiography without contrast media windows. The color render volume window provided the most obvious visualization of the bones (Figures 1 and 2). The serial transverse section of the *Ossa cordis dextrum* had a long elliptical shape within most sections whereas *Ossa cordis sinistrum* had a short elliptical shape and the os aorta had a rounded outline (Figure 3). The sagittal CT detected that the os aorta had an oval at outline, whereas the *Ossa cordis dextrum* and sinistrum were more rounded (Figure 4).

**FIGURE 5:**
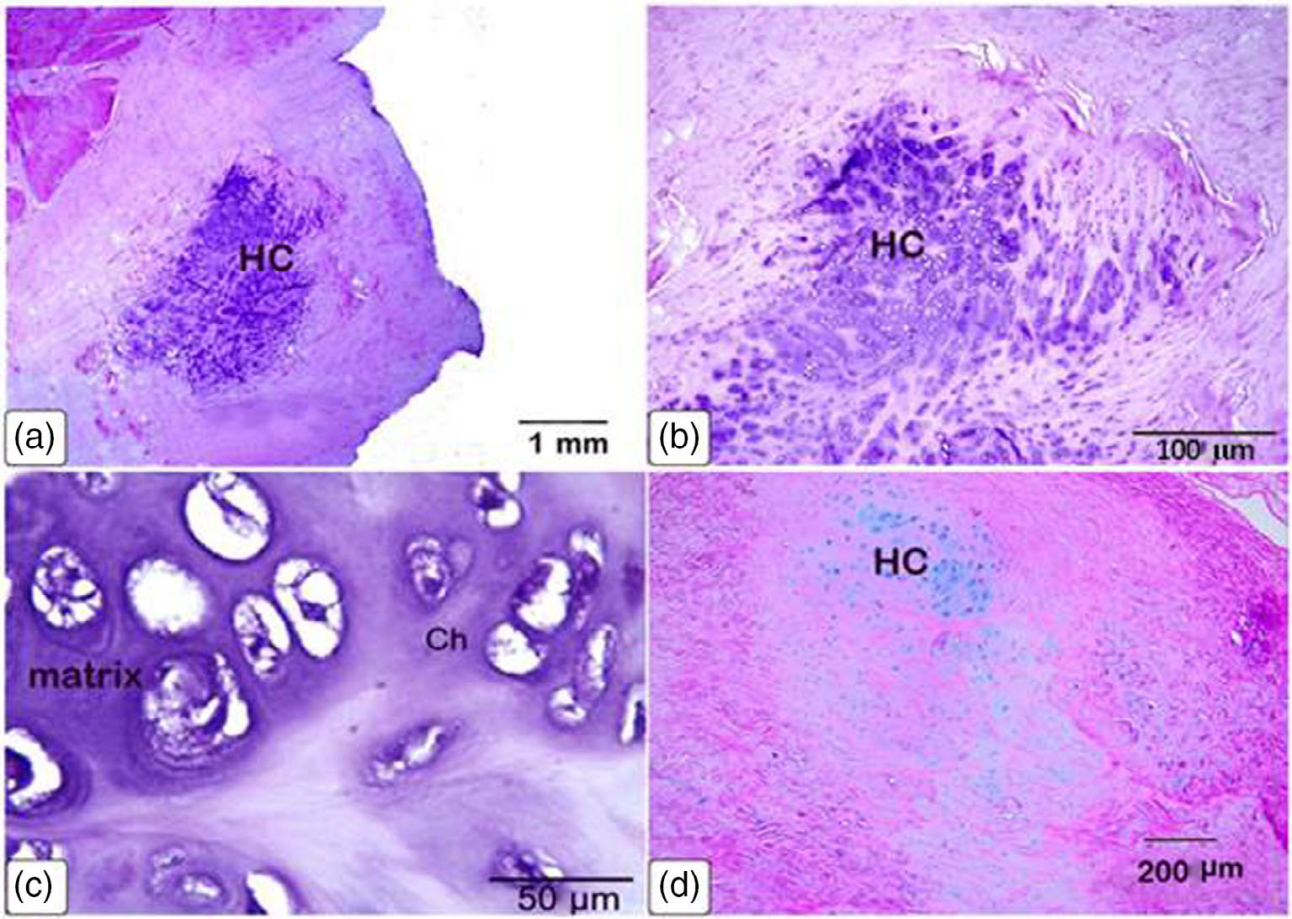
Light photomicrographs of cartilage on the outer surface of *Ossa cordis dextrum* surface. (a–c) H&E stain, (d) Alcian blue pH 2.5. The hyaline cartilage (Hc) and chondrocytes (Ch) on the outer surface of the *Ossa cordis* were surrounded by cartilage matrix (blue in color)

**FIGURE 6:**
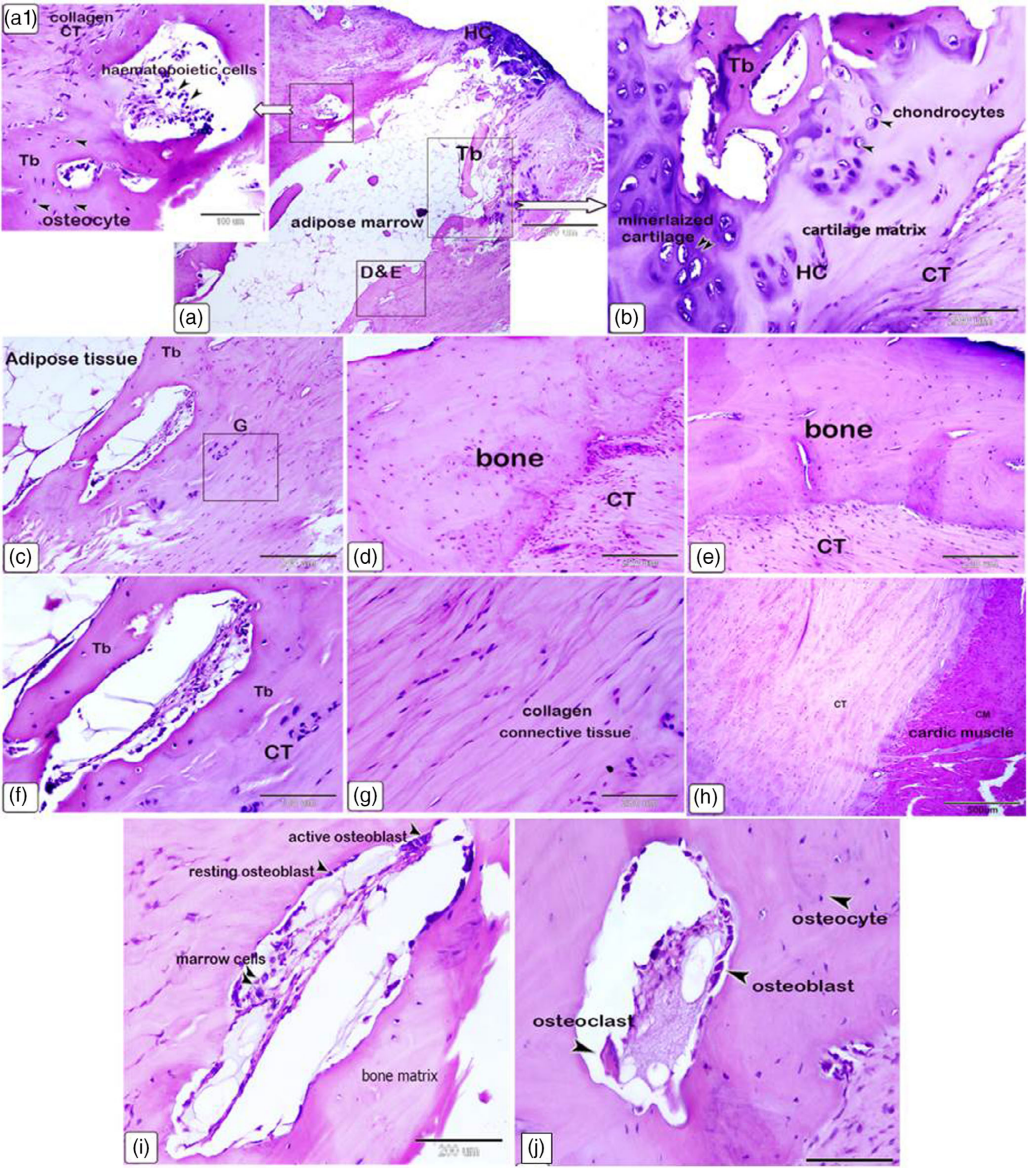
Light photomicrographs of *Ossa cordis*. (a–h) *Ossa cordis dextrum* and associated hyaline cartilage, (i, j) *Ossa cordis sinistrum*. Features include the cardiac muscle (CM) and collagen connective tissue (CT) surrounding the *Ossa cordis* containing trabeculated bone (Tb), containing osteocytes, osteoblasts, osteoclasts, and adipose marrow within the *Ossa cordis*. The cartilage associated with the *Ossa cordis* was characterized by the presence of chondrocytes surrounded by a blue stained cartilage matrix, with the higher intensity staining indicating mineralized cartilage. (h, e) Stain. Scale bars represent a = 500 μm; a1, f = 100 μm; b, c, d, e, g, i, j = 200 μm

**FIGURE 7:**
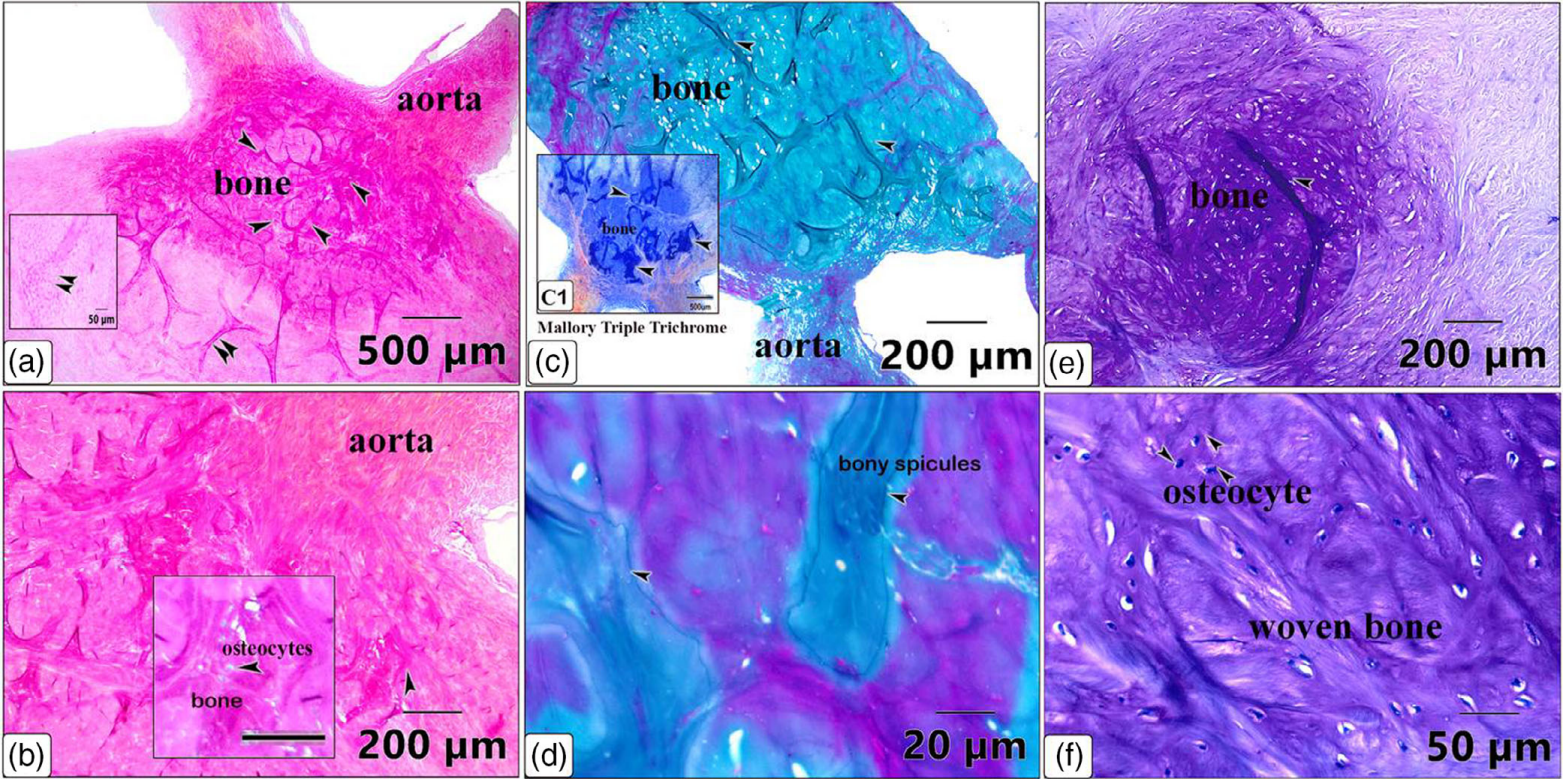
Light photomicrographs of the os aorta located at the start of the ascending aorta. (a, b) Safranin stained bone matrix (pink) with osteocytes (arrowheads) within the lamellar bone. The double arrowheads indicate aggregations of stromal cells. (c, d) Collagen fiber staining using Crossman's and (c1) Mallory's trichrome. The bone is indicated by the arrowheads, the matrix was stained blue using Mallory's and green by Crossman's trichrome. (e, f) Toluidine blue stained the os aorta including trabecular bone and osteocytes (arrowheads) amid the randomly organized collagen fibers and woven bone formation

Histological examinations showed that the large os cordis dextrum in each heart contained trabecular bone, bone marrow, hemopoietic cells and sites of ossification (Figures 5 and 6a,h). Osteocytes, active and resting osteoblasts, and osteoclasts were all present within the trabeculated bone of the *Ossa cordis sinistrum* and *dextrum* (Figure 6). Hyaline cartilage and chondrocytes were observed throughout the tissue but markedly on the outside of the *Ossa cordis* (Figures 5 and 6a–h). In contrast, the smaller *Ossa cordis sinistrum* contained only trabecular bone in each of the three hearts, that were characterized by the presence of osteocytes, active and resting osteoblasts, and osteoclasts (Figure 6i,j). An aggregation of osteocytes and trabecular bone was detected around the ascending aorta, thus indicating the presence of an os aorta (Figure 7). Safranin O stained exhibited a pink color across the bone matrix (Figure 7a,b). Collagen fibers in the bone matrix were stained blue by Mallory's and green by Crossman's trichrome (Figure 7c,d). Toluidine blue showed that the collagen fibers were arranged in a random pattern (Figure 7e–f).

## 4 DISCUSSION

Computed tomography is a noninvasive imaging modality that can be used for the evaluation of cardiac structure in addition to calcified structures. The present study showed several imaging types but additionally detected the *Ossa cordis dextrum* and *Ossa cordis sinistrum*, in addition to os aorta, in the camel, a novel finding for any species.

Cardiac computed tomography in people is achieved with contrast media to evaluate the normal structure and function of the heart and its vessels. On other hand non‐contrast CT is mainly designed for revealing calcification in the coronary artery (Rumberger, 2010). Ossification within the heart is difficult to detect using echocardiography and radiograph but relatively easy of exposure of bone or mineralized cartilage by micro‐CT (Moittié et al., 2020). Recent investigations using computed tomography to identify the *Ossa cordis* in ovines, detected only a single os cordis dextrum (Massari et al., 2021). The present study has shown CT methods, which work even with a relatively large heart, when identifying even small *Ossa cordis* and os aorta.

Previous camel publications stated the presence of only one os cordis within the heart, whereas the present research discovered both *Ossa cordis sinistrum* and dextrum. In 1954 the presence of a triangular shaped os cordis in camels, located to the right of the aortic fibrous ring was published (Hegazi, 1954). More recently the single os cordis dextrum was confirmed as a large, elongated bone embedded bedded within the heart wall to which cardiac muscle was attached (Balah et al., 2014). It was described as substantially within the aortic ring; especially within the adjacent area between the aorta and therefore covered the left and right parts of the heart. It was also positioned within the atrioventricular plane near the junction of the interatrial and interventricular septa of the camel heart and increasing anteriorly into the atrioventricular valve rings. Karkora stated the presence of an elongated bone, measuring 2 cm by 0.5 cm in the right fibrous trigone (Karkora, 1989) and Nawal and colleagues confirmed a singular os cordis within the camel (Nawal et al., 1998). In contrast, another publication presented a *Cartilago cordis*, but not an os cordis in the camel (Smuts & Bezuidenhout, 1987). Other publications, primarily presenting dromedary camel heart morphology in respect to the coronary vessels and cardiac conduction system, also stated the presence of a single os cordis (Balah et al., 2014; Ghonimi et al., 2014).

The discovery of more than one *Ossa cordis* within the same animal does however have precedence in other species. Despite the variability observed relating to *Ossa cordis* presentation, the structure is a relatively a normal element within the ruminant heart. Sheep commonly have *Ossa cordis dextrum* and occasionally have *Ossa cordis sinistrum*, especially in older animals (Frink & Merrick, 1974). The presence of an os cordis sinistrum located near the junction of the interatrial and interventricular septa, extending to the ring of the right atrioventricular valve ring, measuring about 1 cm in length has been published in relation to cattle (James, 1965). However, later research showed a larger os cordis dextrum triangular with two ramus in the caudal border in Holstein, elongated in Iranian cattle and a smaller os cordis sinistrum which was an inconsistent, irregular shape (Pour, 2004). An os cordis sinistrum, in addition to an os cordis dextrum, was highlighted within buffalo, in addition to fibrocartilage encircling the origin of aorta (Daghash & Farghali, 2017). Interestingly, in the camel specimens in the present study, aggregations of osteocytes were visualized in the ascending aorta, not fibrocartilage. Sheep have also presented with none, one or two *Ossa cordis*. From a sample size of 50 per species, 52% of sheep and 44% of goats had an os cordis dextrum. These had an average length and thickness of 18.10 and 2.30 mm respectively in sheep, and 16.99 and 2.25 mm in goats (Mohammadpour, 2007). Other publications have also reported a single os cordis in sheep, with its location reported as within the fibrous ring in both sheep and goats (Massari et al., 2021; Tipirdamaz, 1987). Interestingly, the Tibetan‐sheep has two *Ossa cordis dextrum* in the right fibrous ring, one triangular in shape measuring 76 ± 0.21 mm in length and the other one averaging 0.13 ± 0.24 mm long (Anguo & Chongcun, 1991). In contrast, another study located a single os cordis dextrum deep in the atrial septum, adjacent to the atrioventricular node, and occasionally found an os cordis sinistrum in the left atrioventricular ring in sheep (Frink & Merrick, 1974). It is of interest that *Ossa cordis* have not yet been reported, despite extensive cardiac explorations, in the closely related alpaca (*Vicugna pacos*) and llama (Lama glama), which also resides in the Artiodactyla order, *Tylopoda* suborder, within the *Camelidae* family (Mattoon et al., 2001; Perez et al., 2018). This is especially interesting given that the Old World camels and New World camels generally have hearts more similar to each other than *Ruminantia*, yet many ruminants have *Ossa cordis* (Best et al., 2022; Perez et al., 2018).

A *Cartilago cordis* was not discovered within any of the five camels presented within the present study; however, the histological findings showed that cartilaginous tissue (hyaline cartilage and chondrocytes) was present in association with *Ossa cordis dextrum*. The presence of trabecular bone alongside cartilage was also reported in camels previously (Balah et al., 2014), whereas Smuts and Bezuidenhout (1987) mentioned only *Cartilago cordis* within their camel specimens, not *Ossa cordis*. Other species, such as the otter, chimpanzee (Moittie et al., 2020) have previously shown the presence of either *Ossa cordis* or a *Cartilago cordis* in differing animals, showing that both are possible within the same species, yet both occurring simultaneously have not yet been discovered within the same individual animal. Some research has also shown that the *Ossa cordis* can with associated cartilage such as in the otter, goat and chimpanzee (Egerbacher et al., 2000; Moittie et al., 2020; Tipirdamaz, 1987). The evidence from the present research shows that another species, the camel, has cartilage tissue associating with *Ossa cordis*, which further supports our theory that ossification may initiate from cartilaginous tissue.

The general use of a trichtome to identify bone matrix in os aorta was previously described by (Bancroft & Gamble, 2008). The os aorta in the camels in the present study showed that the bone matrix tested negative for Safrainin O, which denotes a lack of glycosaminoglycan, which gives cartilage its orange‐red color using this stain. It has also been reported that the presence of immature bone or woven bone in the os aorta indicates recent bone remodeling (Bancroft & Gamble, 2008), a feature shown in the camel os aorta in the present study.

## 5 CONCLUSION

A novel discovery of one os cordis dextrum, two *Ossa cordis sinistrum* and one os aorta were discovered within three of five camel hearts (male, aged 13 years). Two other hearts (also male, 13 years old) showed a single os cordis dextrum. The os cordis dextrum was located in the upper part interventricular septum, near its junction with the atrium, and passed transversely through the structure in an elongated rectangular shape. The cranial part of the os cordis dextrum was wider and ossified, whereas the narrow caudal part contained both trabecular bone and cartilaginous tissue. The smaller *Ossa cordis sinistrum* were located in different locations, with one within the right fibrous ring and the other in the interventricular septum. The os aorta was in the wall of the ascending aorta. This research represents the first time a camel heart has used CT scanning and histology to investigate *Ossa cordis*. The presence of an os cordis dextrum, *Ossa cordis sinistrum* and os aorta within the camel is a novel discovery within any species.

## AUTHOR CONTRIBUTIONS

**Samir A.A. El‐Gendy:** Conceptualization; data curation; formal analysis; funding acquisition; investigation; methodology; project administration; resources; software; validation; visualization; writing – original draft; writing – review and editing. **Mohamed A.M. Alsafy:** Conceptualization; data curation; formal analysis; funding acquisition; investigation; methodology; project administration; resources; software; validation; visualization; writing – original draft; writing – review and editing. **Catrin S. Rutland:** Conceptualization; formal analysis; funding acquisition; investigation; validation; visualization; writing – original draft; writing – review and editing. **Samar M. Ez Elarab:** Data curation; formal analysis; investigation; methodology; validation; visualization; writing – original draft; writing – review and editing. **Hanan H. Abd‐Elhafeez:** Data curation; formal analysis; investigation; methodology; validation; visualization; writing – original draft; writing – review and editing. **Basma Kamal:** Data curation; formal analysis; investigation; methodology; validation; visualization; writing – original draft; writing – review and editing.

## FUNDING INFORMATION

The authors would like to thank the Faculty of Veterinary Medicine, Alexandria University and the School of Veterinary Medicine and Science, University of Nottingham.

## CONFLICT OF INTEREST

None of the authors has any financial or personal relationships that could inappropriately influence or bias the content of the paper.

## Data Availability

The data that support the findings of this study are available from the corresponding author upon reasonable request.
